# Visitation Access to U.S. Nursing Homes: An Analysis of Facility Locations, Ratings, and Disparities

**DOI:** 10.1093/geroni/igaa057.375

**Published:** 2020-12-16

**Authors:** Mary Helander

**Affiliations:** Syracuse University, Syracuse, New York, United States

## Abstract

Research findings suggest that family visits to nursing home residents are important for countering depression, increasing residents’ well-being and quality of life (Miller, 2019; Durkin et al., 2014), maintaining physical function (Shankar et al., 2017), and improving general health (Parmenter et al., 2012). Presence by family can directly impact a resident’s care quality, since family members may take a role in monitoring their older relative’s status (Miller, 2019). Unfortunately, regular family visits to nursing homes may be difficult, or impossible, due to challenges that include distance, travel time, lack of transportation, and cost (Fields et al., 2019) (Miller, 2019). These same challenges may translate to socio-economic barriers for families, eliminating long-term-care as an option for older relatives (Ferraro et al., 2017), (Angel and Berlinger, 2018). This paper considers the issue of nursing home visitation access and examines related disparities through spatial and demographic analysis of 15,000+ US facilities monitored by the Centers for Medicare the Medicaid Services. Mathematical models are used to analyze facility and population data, using access measures adapted from the geography discipline (Lou and Wang, 2003; Paez et al., 2019). Analysis explores whether higher rated nursing homes are more likely to be closer to affluent populations, and whether socioeconomic status is a significant factor in overall access. Analysis reveals patterns of access disparity with respect to nursing home ratings and geographies. For example, proximity to higher rated facilities increases monotonically with median household income. Specific policy recommendations are discussed.

